# Acidic pH promotes degranulation and reduces oxidative stress in human primary neutrophils

**DOI:** 10.17179/excli2026-9092

**Published:** 2026-07-13

**Authors:** Maximilian Göbel, Yangfan Li, Melike Tombaz, Filiz Sahin, Andreas K. Nussler, Sabrina Ehnert

**Affiliations:** 1 Siegfried Weller Research Institute, BG Unfallklinik Tuebingen, Department of Trauma and Reconstructive Surgery, Eberhard-Karls University Tuebingen, Schnarrenbergstraße 95, D-72076 Tuebingen, Germany

**Keywords:** neutrophils, pH, degranulation, exocytosis, ROS, wound healing, oxidative stress

## Abstract

Chronic wounds represent an increasing global health burden for both patients and healthcare systems. These non-healing lesions frequently result in amputation and are particularly prevalent among individuals with diabetes. Hallmarks of chronic wounds include a prolonged inflammatory phase characterized by sustained neutrophil infiltration, excessive formation of neutrophil extracellular traps (NETs), and an elevated alkaline tissue pH. Emerging evidence suggests a link between pH and wound healing, with acidic conditions being more favorable for tissue repair. Here, we investigated the pH-dependent modulation of primary neutrophil functions, including viability, reactive oxygen species (ROS) production, NET formation, and exocytosis. Neutrophils cultured in media with pH values ranging from 6.1 to 8.4 exhibited no significant differences in viability. In contrast, acidic conditions suppressed ROS production and NET formation in a pH-dependent manner, whereas alkaline conditions caused responses comparable to those observed at physiological pH (7.4). Furthermore, levels of antioxidant enzymes were markedly increased under acidic conditions following phorbol 12-myristate 13-acetate (PMA) stimulation. Notably, alkaline pH promoted granule polarization in neutrophils, thereby impairing exocytosis. In contrast, acidic conditions (pH < 7.0) reduced granule polarization and enhanced the release of granule-associated proteins upon PMA stimulation. Pharmacological inhibition of degranulation confirmed that these effects were mediated by neutrophil exocytosis. In summary, extracellular pH critically modulates neutrophil effector functions and may contribute to the distinct healing dynamics observed in acute versus chronic wounds.

See also the graphical abstract(Fig. 1).

## Introduction

Diabetes mellitus is the metabolic disease with the most rapidly increasing incidence and prevalence worldwide (Sun et al., 2022[45]). Chronic hyperglycemia is the hallmark of this disorder and gives rise to numerous comorbidities. One major complication is diabetic foot ulcer (DFU), which affects approximately 19-34  % of patients with type 2 diabetes (Armstrong et al., 2017[1]) and frequently leads to amputation of the affected limb. Chronic wounds generally represent a substantial burden or patients, and their pathogenesis is multifactorial and not yet fully understood. Wound healing is a tightly regulated process consisting of four overlapping phases: hemostasis, inflammation, proliferation, and remodeling (Pena and Martin, 2024[33]). Chronic wounds are characterized by a prolonged and dysregulated inflammatory phase, which impairs progression to subsequent stages of healing (Schilrreff and Alexiev, 2022[41]). The inflammatory phase is shaped by various immune cells, particularly macrophages and granulocytes. Neutrophils act as first responders after tissue injury and rapidly migrate to the affected site to combat invading pathogens (Wilgus et al., 2013[48]). To fulfill this function, neutrophils deploy a broad repertoire of antimicrobial strategies. In addition to phagocytosis, the release of antimicrobial proteins and cytokines is a key defense mechanism. This process, termed exocytosis or degranulation, is essential for innate immune responses (Faurschou and Borregaard, 2003[13]).

Neutrophils contain several classes of cytoplasmic granules distinguished by their protein composition: primary (azurophilic), secondary (specific), and tertiary (gelatinase) granules (Borregaard and Cowland, 1997[4]). The tetraspanin protein CD63 is localized to the membrane of primary granules (Cham et al., 1994[9]), which store characteristic enzymes such as myeloperoxidase (MPO) and neutrophil elastase (NE). Specific granules contain antimicrobial proteins including cathelicidin (LL-37) and lactoferrin, as well as the cytochrome b_558_ subunit of nicotinamide adenine dinucleotide phosphate (NADPH) oxidase (NOX), which is responsible for reactive oxygen species (ROS) production (Faurschou and Borregaard, 2003[13]). Tertiary granules contain matrix metalloproteinases (MMPs), such as MMP2 and MMP9, and are therefore also referred to as gelatinase granules (Mollinedo et al., 1991[29]; Rorvig et al., 2013[39]). Upon activation through receptor engagement (for review see Futosi et al., 2013[14]) or chemical stimuli such as phorbol 12-myristate 13-acetate (PMA), neutrophils initiate diverse defense mechanisms, including intracellular antioxidant pathways that protect against oxidative stress. Enzymes such as glutathione peroxidase 1 (GPX1), glutathione synthetase (GS), and superoxide dismutase 1 (SOD1) play central roles in these processes (Brinkmann et al., 2025[5]; Damascena et al., 2022[11]; Pietarinen-Runtti et al., 2000[34]). GPX1 catalyzes the reduction of H₂O₂ to H₂O (Handy and Loscalzo, 2022[16]) using reduced glutathione (GSH) as a co-substrate, which is synthesized by GS from cysteine. SOD1 converts superoxide anions (O₂⁻) into H₂O₂, providing substrate for GPX1 (Kellner et al., 2017[21]). Beyond classical functions such as ROS production, degranulation, and phagocytosis, Brinkmann et al. identified an additional neutrophil defense mechanism in 2004 termed neutrophil extracellular trap (NET) formation. NETosis is a specialized form of cell death characterized by the release of chromatin decorated with citrullinated histones and antimicrobial proteins into the extracellular space, where it traps and neutralizes pathogens (Brinkmann et al., 2004[6]). Over the past decade, excessive NET formation has been implicated as a detrimental factor in diabetic wound healing (Sabbatini et al., 2021[40]; Wong et al., 2015[49]). NET components impair stem-cell viability, inhibit fibroblast proliferation and collagen synthesis, and disrupt angiogenesis by endothelial cells (Linnemann et al., 2022[24]; Zhu et al., 2021[50]). Antioxidant systems have emerged as key regulators of inflammation and neutrophil function, and Brinkmann et al. recently demonstrated that SOD1 is a critical mediator of NET formation through its regulation of ROS levels (Brinkmann et al., 2025[5]).

A defining feature of chronic wounds is their elevated alkaline pH (pH > 7) compared to acute and normally healing wounds (Schneider et al., 2007[42]; Tricou et al., 2024[46]; Wallace et al., 2019[47]). In contrast, physiological blood pH is maintained within a narrow range of 7.35-7.45. The mechanisms underlying wound alkalization remain poorly understood. In acute wounds, tissue pH initially decreases due to metabolic alterations in injured cells, leading to increased lactate production (Haller et al., 2021[15]; Wallace et al., 2019[47]). Acidic wound environments have been associated with reduced bacterial growth and accelerated wound closure (Malu et al., 2016[28]; Sim et al., 2022[43]). Notably, two recent studies reported enhanced NET formation under alkaline conditions (Behnen et al., 2017[3]; Khan et al., 2018[22]), raising the question of how pH modulates neutrophil defense mechanisms. The present study therefore aimed to investigate the effects of extracellular pH on the balance between ROS production, NET formation, and degranulation in primary human neutrophils.

## Methods

### Preparation of pH-media

RPMI1640 without phenol red (Sigma, Taufkirchen, Germany) was used to culture primary neutrophils. The medium contains no supplements and was adjusted to the desired pH by adding HCl or NaOH directly before the experiments. Adjustment was done at 37 °C. 

### Isolation of human primary neutrophils

Human primary neutrophils were isolated from healthy volunteers aged between 21 and 44 years (median: 29 ± 4.8 SD; equal distribution of males and females) (Ethical Vote: 666/2018BO2). Venous blood was collected in EDTA-tubes and immediately layered onto Lympholyte-poly density gradient medium (CEDARLANE, Burlington, USA), for isolation by density gradient centrifugation at 500 x g for 40 minutes (no brake). The neutrophil layer was collected and washed twice with Phosphate buffered saline (PBS) as described (Linnemann et al., 2022[24]). Cells were resuspended in RPMI1640 medium and counted by trypan blue nuclear extension method. Until use, cells were stored in RPMI1640 without phenol red. 

### Viability testing of pH-cultured neutrophils

Isolated neutrophils were cultured in different pH media (pH 6.1-8.4), at a concentration of 0.5 x 10^6^/mL, for up to three hours at 37 °C and 5 % CO_2_. After cultivation, the 96-well plate was centrifuged (600 x g; 10 minutes) and the medium was removed. Calcein-AM solution was added in a concentration of 1:1000 (2 µM) in PBS, and incubated for 30 minutes at 37 °C in the dark. Ethidium Bromide was added (2 µM) during the last 5 mins of incubation. After the staining solution was replaced by PBS, fluorescent intensity (λex/em = 485/520 nm for Calcein and λex/em = 544/490 nm for ethidium bromide) was quantified with a plate-reader (FLUOstar Omega, BMG Labtech, Ortenberg, Germany). Afterward, fluorescence microscopy images were taken by the EVOS FL (Live Technologies, Darmstadt, Germany) at a 10x magnification (Linnemann et al., 2022[24]).

### Intracellular pH measurement

Intracellular pH was determined by a single pH detection kit (CytoCHECK SPAchip® green single detection kit, S-001-PHG, a4cell nanodevices, OLS, Bremen, Germany). Only changes in acidic pH were measured by following the manufacturer's protocol. Briefly, three different pH levels (5.4, 6.4 and 7.4) were used to develop a calibration line, and 2.5 x 10^6^ chips were used per pH-condition. Isolated neutrophils were mixed with pH-sensing chips (ratio 1:2) and incubated for 3 h. Fluorescence intensity was measured by a Sysmex flow cytometer (λex/em = 488/536 nm for GFP) (FlowCube8, Sysmex, Hamburg, Germany). Analysis of flow cytometry data was performed using the FCS Express 5 RUO software (De Novo Software, Pasadena, USA).

### Determination of ROS production in pH-cultured neutrophils

ROS production was quantified by 2´,7´- dichlorodihydrofluorescein diacetate (DCFH-DA) assay. Cells were seeded in a concentration of 1 x 10^6^/mL as described previously (Linnemann et al., 2023[25]) and resuspended in pH-adjusted medium (pH 6.1-8.4). The 96-well plate was cultured for 30 minutes or three hours. Then, the pH medium was replaced by DCFH-DA solution (20 µM DCFH-DA). After 30 minutes of incubation, cells were washed once before pH medium with or without PMA (100 nM) was added. Fluorescence intensity (λex/em = 485/520 nm) was quantified by a microplate reader (FLUOstar Omega, BMG Labtech, Ortenberg, Germany) over a time range of 30 minutes, measured every two minutes; the results are shown as the slope of the fluorescence intensity.

### SYTOX Green assay of pH-cultured neutrophils

NET formation was analyzed by SYTOX Green assay. For this, cells were seeded at a concentration of 2 x 10^5^/mL in different pH-adjusted (6.1-8.4) media. SYTOX Green dye was added in a concentration of 1 µM. To induce NET formation, protein kinase C activator PMA was used at a concentration of 100 nM. Cells were incubated for 18 hours at 37 °C and 5 % CO_2_ in the microplate reader (CLARIOstar^Plus^, BMG Labtech, Ortenberg, Germany), and fluorescence intensity (λex/em = 488/535 nm) was quantified every 30 minutes. After three hours, microscopy images were taken by EVOS FL (Live Technologies, Darmstadt, Germany). AUC of SYTOX Green intensity was determined and normalized to total DNA release (Triton-X-100 control) (Linnemann et al., 2020[26]).

### Exocytosis determination of pH-cultured neutrophils

Exocytosis was measured with neutrophils in different pH media ± 100 nM PMA. Due to the increased cell amount required, only 3 different pH conditions were tested at the same time (always including pH 7.4 as reference). All isolated cells were distributed equally to the different conditions. To prove exocytosis, a degranulation inhibitor (Nexinhib20, Cayman Chemical, Ann Arbor, USA) was added at a final concentration of 10 µM. After 3 hours of incubation, the supernatant was collected and centrifuged at 17.000 x g at 4 °C. The supernatant was stored at -80 °C until use. The release of granular proteins characteristic of all three types of neutrophil granules was analyzed by the Dot Blot method. 100 mL of each supernatant were transferred to a nitrocellulose membrane using a Dot Blot device (T790.1, Carl Roth, Germany). Membranes were blocked with 5 % Bovine serum albumin (BSA, Roth, 8076.4) in Tris buffered saline with Tween-20 (0.1 %) (TBS-T) for one hour and incubated with primary antibodies overnight (MPO 1:500, sc-52707 Santa Cruz; NE 1:500, sc-55549, Santa Cruz; LL-37, 1:500, sc-16670, Santa Cruz; MMP9, 1:500, sc-21733; CCL-2 1:500, sc-377082, Santa Cruz, Biotechnology Europe, Heidelberg, Germany; Interleukin-1β 1:1000, 12703, Cell Signaling Technology, Leiden, Netherlands). Primary antibodies were detected by goat-anti-mouse- and goat-anti-rabbit-antibodies coupled with Horseradish peroxidase (HRP) (Santa Cruz Biotechnology Europe, Heidelberg, Germany). Following an incubation for 1 minute with ECL-solution (2.5 mM Luminol, 0.4 mM p-Coumaric acid, 0.06 % H_2_O_2_ in 100 mM TRIS pH 8.5) signal intensities were captured with the ChemiDoc from Intas (INTAS, Göttingen, Germany). Signal intensities were quantified using the ImageJ 1.54 (NIH, USA) software (Haussling et al., 2019[17]).

### Immunostaining of pH-cultured neutrophils

Cells were seeded in a concentration of 3 x 10^5^ cells/mL in different pH media and stimulated ± 100 nM PMA as described earlier (Linnemann et al., 2020[26]). After different time points (0.5 h, 1.5 h, and 3.0 h), cells were fixed by 4 % paraformaldehyde (PFA) for 30 minutes. 0.1 % Triton-X-100 was used to permeabilize the cells for 5 minutes. The fixed neutrophils were washed three times with PBS, and unspecific binding sites were blocked by 5 % BSA in PBS. Primary antibodies against CD63 (1:200, sc-59286, Santa Cruz Biotechnology) and MPO (1:200, sc-52707, Santa Cruz) were incubated overnight at 4 °C. After washing three times with PBS, slides were incubated with AlexaFlour-488-coupled secondary antibodies (2 µg/mL) and Hoechst (2 µg/mL) for two hours. Then, slides were air-dried and cover slides were added by mounting medium before capturing fluorescent images with a Logos Biosystems microscope (CELENA X, Villeneuve d'Ascq, France).

For quantification of the polarization of primary granules, CD63-EGFP (20x magnification) images were analyzed, and cells that show a polarized CD63 (bright, focused, and next to the nucleus, not at the cell membrane) signal were counted manually and normalized to the DAPI relative fluorescence intensity. Moreover, the relative fluorescence intensity of the CD63-EGFP was measured. For image analysis, ImageJ 1.54 (NIH, USA) was used. 

### Western blot

The isolated neutrophils were resuspended in media with different pH values and seeded into a 6-well plate. All isolated cells were distributed evenly across the different conditions. 100 nM PMA was used as the activator, and the cells were incubated for three hours. After the incubation period, RIPA buffer (containing pepstatin, leupeptin, phenylmethylsulfonyl fluoride, sodium fluoride, and sodium orthovanadate) was used to obtain the cell lysate. Protein quantification was performed using the Micro-Lowry method. 50 µg of protein was loaded onto a sodium dodecyl sulfate (SDS)-polyacrylamide gel (10 %). Transfer was performed using a semi-dry blot (Semi-Dry Blotter, Carl Roth, Karlsruhe, Germany). Nitrocellulose membranes (Amersham Protran, 0.2 µm, Cytiva, Freiburg, Germany) were blocked with 5 % BSA and incubated overnight at 4°C with primary antibodies (glutathione synthetase, 1:1000, ab133592, glutathione peroxidase, 1:1000, ab22604, Abcam Limited, Cambridge, United Kingdom, superoxide dismutase, 1:1000, 4266, Cell Signaling Technology, Leiden, Netherlands) (Ehnert et al., 2017[12]). For normalization, total protein content was determined using Ponceau staining. Standard loading controls such as GAPDH and HPRT showed pH-dependent regulation. HRP-conjugated secondary antibodies were incubated for 2 hours at room temperature. The membranes were developed with ECL solution, and the signals were quantified using ImageJ. 

### Statistical analysis

Each experiment was done in at least three biological replicates (N≥3) and technical duplicates (n≥2). The exact number of biological and technical replicates is given on the figure legends, whereby “N” denotes biological replicates and “n” technical replicates. Statistical analysis was performed by PRISM 8 (GraphPad Prism 8.0.1) software. Data sets were compared using the non-parametric Kruskal-Wallis test, followed by Dunn´s multiple comparison test, or by 2-way ANOVA, followed by multiple comparisons. If not otherwise stated, data were normalized and compared to the stimulated pH 7.4, physiological condition. A p < 0.05 was considered statistically significant.

## Results

### Neutrophil viability is not affected by the pH

**Figure 2:**
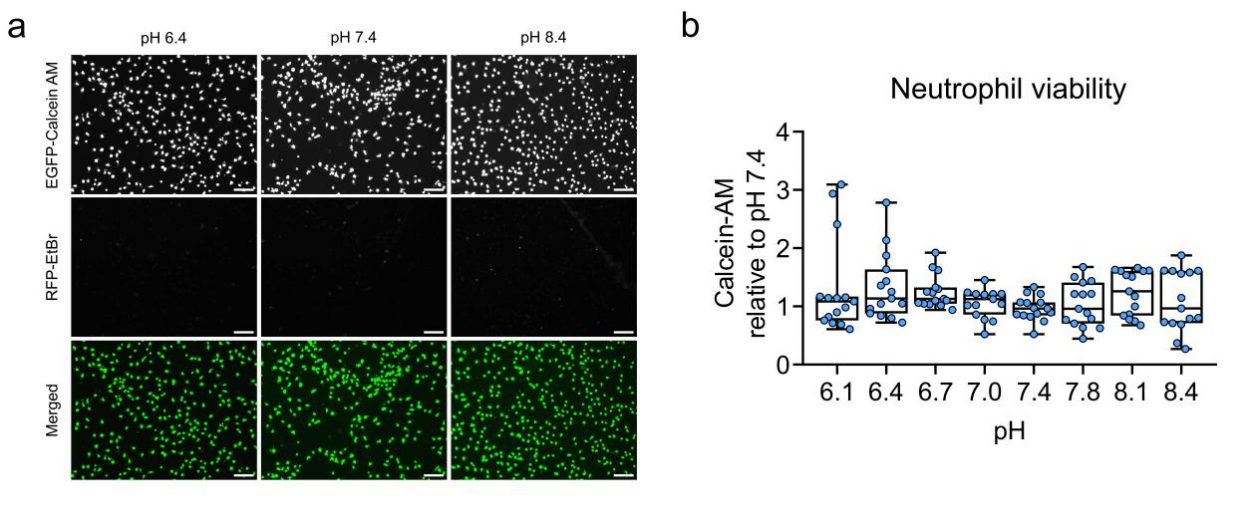
Primary neutrophil viability at different pH. Immediately after isolation, primary human neutrophils were incubated in different pH-media (6.1-8.4; exemplary 6.4, 7.4, and 8.4) for three hours. Living cells were detected by Calcein-AM conversion to Calcein. Dead cells were detected by staining with ethidium bromide. a) Representative fluorescent microscopy images of the live-dead staining. Scalebar: 100 µm. b) Resulting Calcein signals were quantified with a plate-reader. Fluorescent signals were normalized to the signal of pH 7.4, N=5, n=3. Groups were compared by non-parametric Kruskal-Wallis-Test, followed by Dunn´s multiple comparison test. Calcein signals, representing viable cells were comparable (p > 0.05) between all conditions.

First, the effect of different extracellular pH conditions on neutrophil viability was assessed. After three hours of incubation in pH-adjusted media ranging from pH 6.1 to 8.4, primary neutrophil viability was determined by live-dead staining (Figure 2a(Fig. 2)). A pH range between 6.4 and 8.4 did not significantly affect cell viability, as indicated by sustained Calcein-AM conversion and only sporadic staining of dead cells with ethidium bromide (EtBr). Overall, neutrophil morphology and phenotype remained unchanged across the tested pH conditions. Quantification of Calcein fluorescence using a plate reader confirmed these observations (Figure 2b(Fig. 2)).

### ROS production in neutrophils is dependent on the pH

**Figure 3:**
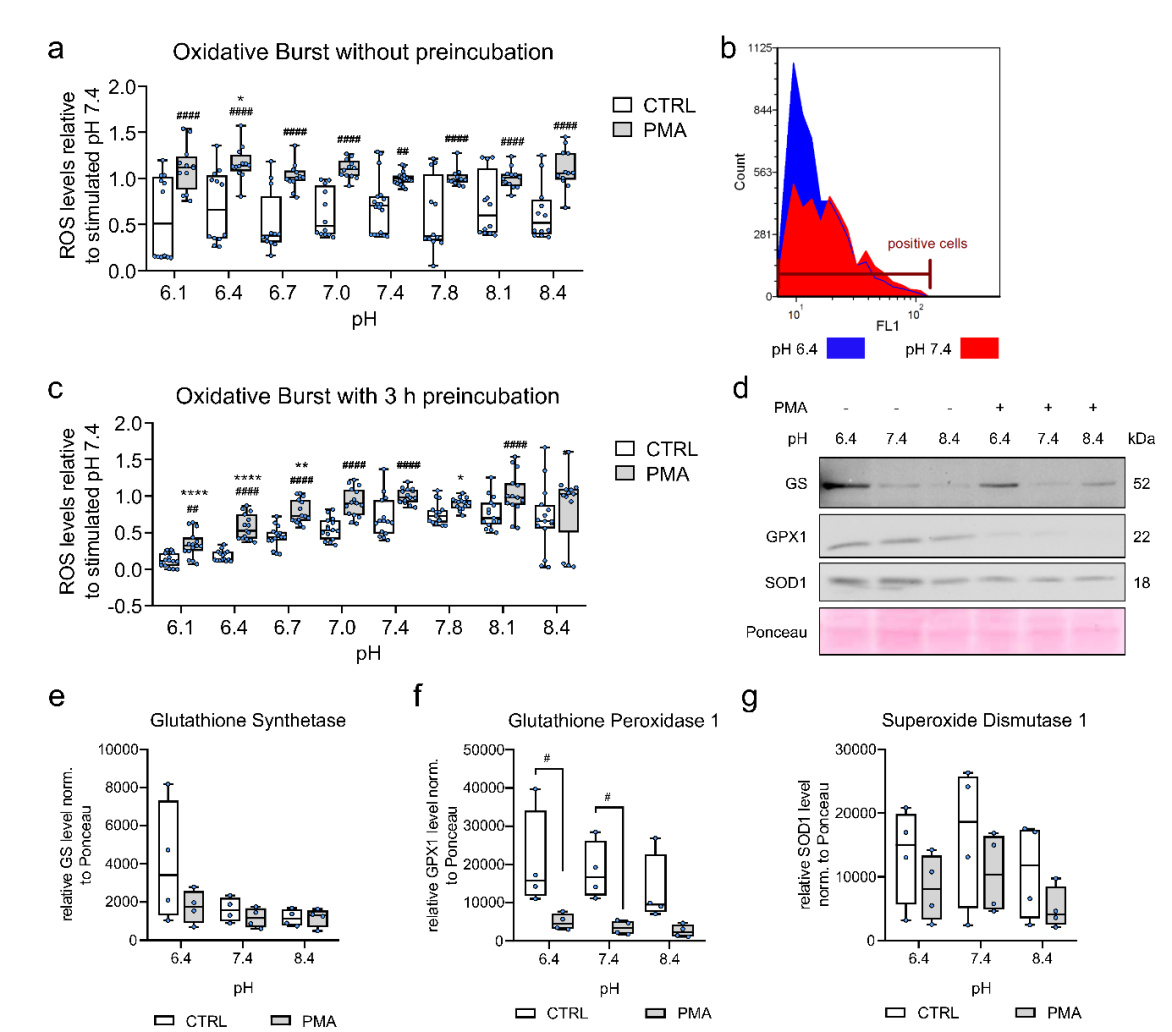
Reactive Oxygen Species (ROS) production of neutrophils is suppressed in acidic pH. ROS production was determined by DCFH-DA-assay. a) When DCFH-DA loaded neutrophils were transferred to different pH-adjusted media no alterations in basal or PMA induced ROS production was detected, which also excludes pH-effects on the assay itself. b) Shift in Fluorescence intensity dependent on the pH (pH 6.4 blue, pH 7.4 red). c) When ROS formation was measured in pH-conditioned (3 hours) neutrophils, the acidic pH range gradually suppressed basal and PMA induced ROS formation. Oxidative burst was induced by 100 nM PMA. N≥5, n=3. d) Representative Blot membranes of pH-cultured neutrophil antioxidative enzymes, including a Ponceau staining for normalization. e-g) Quantification of Glutathione Synthetase, Glutathione Peroxidase 1, and Superoxide Dismutase 1 protein levels in pH-cultured (3 h) neutrophils, showing reduced enzyme level in alkaline pH (N=4, n=1). Groups were compared by two-way ANOVA. *p < 0.05, **p < 0.01, ***p < 0.001, ****p < 0.0001 compared to pH 7.4 and #p < 0.05, ##p < 0.01, ###p < 0.001, ####p < 0.0001 compared basal vs. PMA induced in each pH condition.

ROS generation via the oxidative burst represents an early hallmark of neutrophil activation. To assess the influence of extracellular pH on ROS production, neutrophils were incubated in pH-adjusted media and analyzed using a DCFH-DA assay. Acute changes in extracellular pH did not directly induce ROS production, nor did they acutely alter PMA-induced ROS formation (Figure 3a(Fig. 3)). However, prolonged incubation for three hours under different pH conditions significantly altered both basal ROS levels and the responsiveness to PMA stimulation. Basal ROS production gradually decreased with decreasing pH (Figure 3b(Fig. 3)). Moreover, acidic conditions markedly suppressed PMA-induced ROS generation compared to physiological pH. To investigate potential regulatory mechanisms, antioxidative enzyme expression was analyzed by Western blotting. GS protein levels were increased under acidic conditions and decreased in physiological and alkaline pH ranges (Figure 3d-e(Fig. 3)). In unstimulated cells, GPX1 and SOD1 protein levels were largely pH-independent, although GPX1 expression was lowest under alkaline conditions (Figure 3f-g(Fig. 3)). Upon stimulation with 100 nM PMA, GPX1 levels were significantly reduced across all pH conditions (Figure 3f(Fig. 3)). Similar trends were observed for GS and SOD1, with acidic pH conditions tending to preserve higher enzyme levels following activation (Figure 3e, g(Fig. 3)). To determine whether extracellular pH altered intracellular or intralysosomal pH, neutrophils were analyzed using a pH-sensitive fluorescent probe (SPAchips). Increasing pH was associated with elevated fluorescence intensity (Figure 3b(Fig. 3)). However, only minor shifts in intracellular fluorescence were detected between acidic and alkaline conditions (blue versus red populations), indicating limited intracellular pH changes.

### Acidic pH suppresses NET formation

**Figure 4:**
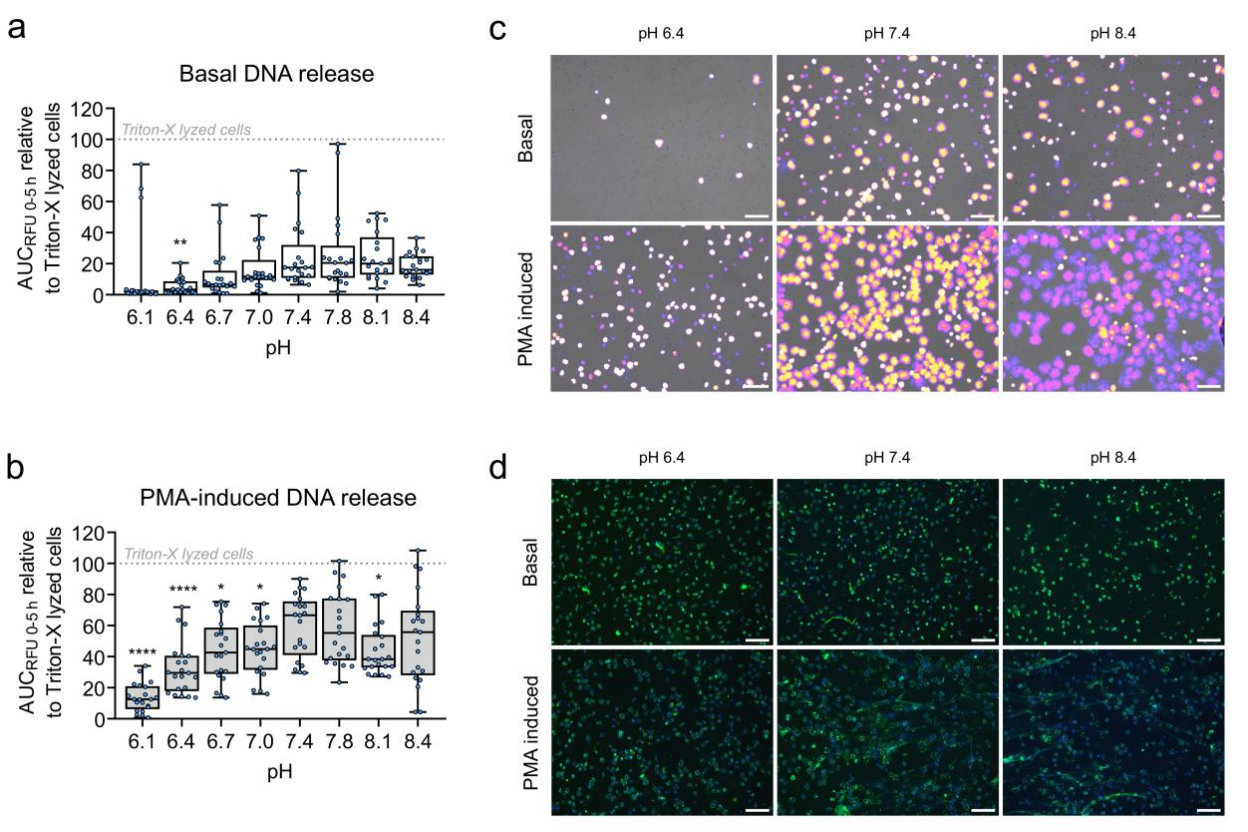
Acidic pH inhibits NET formation. SYTOX Green assay was used to determine DNA release and for NET structure visualization Immunofluorescence staining was performed. a) SYTOX Green stained neutrophils showed a pH-dependent NET release in basal conditions. b) With PMA stimulation this effect was more pronounced. c) Images of SYTOX Green stained cells confirm the plate reader measurement. Scalebar: 100 µm. d) More web-like DNA (Hoechst 33342) structures co-staining with MPO (EGFP) were detected in the neutral and alkaline pH (± 100 nM PMA). Scalebar: 100 µm. N=7, n=3. Data are presented as Box plots with individual data points Groups were compared by 2-way-ANOVA to pH 7.4, *p < 0.05, **p < 0.01, ****p < 0.0001 as indicated.

Because ROS are critical mediators of NETosis (Azzouz and Palaniyar, 2024[2]), the effect of pH on NET formation was examined. NET release was quantified using a SYTOX Green assay under different pH conditions. Under basal conditions, neutrophils released significantly more extracellular DNA at neutral and alkaline pH compared to acidic pH (Figure 4a(Fig. 4)). Following NETosis induction with 100 nM PMA, this difference became more pronounced, with maximal DNA release observed at pH 7.4 (Figure 4b(Fig. 4)). To exclude saturation effects of PMA, the experiment was repeated using a lower PMA concentration (50 nM) at pH 6.4, 7.4, and 8.4, yielding comparable results (Supplementary Figure 1a). Fluorescence microscopy was used to distinguish NETosis from necrosis and revealed diffuse extracellular DNA structures characteristic of NETs (Figure 4c(Fig. 4)). Immunofluorescence staining for MPO and DNA (Hoechst 33342) further confirmed NET formation (Figure 4d(Fig. 4)). While abundant MPO- and DNA-positive NET structures were detected at pH 7.4 and 8.4, these structures were largely absent under acidic conditions (pH 6.4).

### An acidic pH does not affect the basal release of neutrophil granule proteins, but enhances their PMA-induced release 

**Figure 5:**
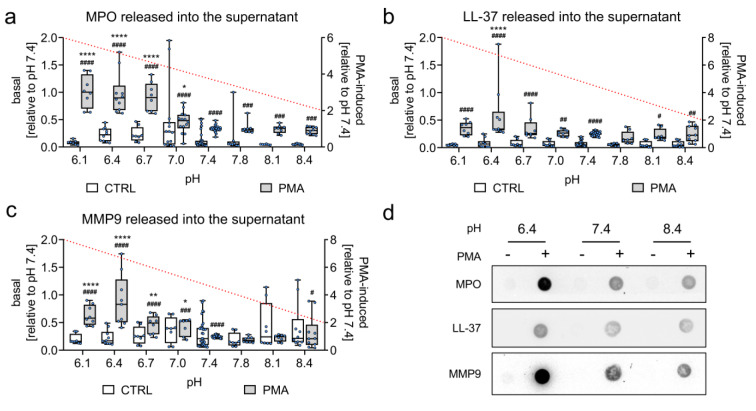
Release of neutrophil granule protein is enhanced in acidic pH. Protein amount was detected by Dot Blot method of neutrophils without PMA stimulation (white boxes - left axis) and 100 nM PMA stimulated (grey boxes - right axis). a) Myeloperoxidase (MPO) as representative of primary granules. b) Release of specific granules was detected by LL-37. c) Tertiary granules represented by Matrix-Metalloprotease 9 (MMP9). d) Exemplary membrane images of the Dot Blot experiments and quantified by ImageJ. N≥3, n=2; Data are presented as Box plots with individual data points Statistical analysis was done by 2-way-ANOVA, while PMA-induced were compared to pH 7.4 respectively *p < 0.05, **p < 0.01, ***p < 0.001, ****p < 0.0001. Differences in each pH from basal to PMA induced are marked by #p < 0.05, ##p < 0.01, ###p < 0.001, ####p < 0.0001 (2-way-ANOVA).

To assess the impact of pH on neutrophil exocytosis, primary neutrophils were cultured in media adjusted to pH 6.1-8.4 under basal conditions and following stimulation with 100 nM PMA (Damascena et al., 2022[11]). After three hours, culture supernatants were collected and granule protein release was quantified by Dot Blot analysis (Figure 5(Fig. 5)). Basal release of granule proteins was minimal and was not significantly influenced by extracellular pH (white boxes). In contrast, PMA stimulation (light grey boxes) led to a marked, pH-dependent increase in granule release. Neutrophils cultured under acidic conditions (pH 6.1-6.7) released two- to four-fold higher levels of primary granule proteins, such as MPO and NE (Supplementary Figure 1b), compared to cells maintained at physiological pH (Figure 5a(Fig. 5)). The release of LL-37, representing specific granules, was significantly affected by pH only at pH 6.4 (Figure 5b(Fig. 5)). Similarly, MMP9 release from tertiary granules exhibited a pH-dependent pattern comparable to that of primary granule markers, with maximal release under acidic conditions. Alkaline pH did not significantly alter PMA-induced granule release compared to physiological pH. Analysis of cytokine release at pH extremes (6.4, 7.4, and 8.4) further revealed elevated secretion of CCL2 and a trend toward increased IL-1β release under acidic conditions (Supplementary Figure 3).

### Neutrophil exocytosis is enhanced in acidic pH

**Figure 6:**
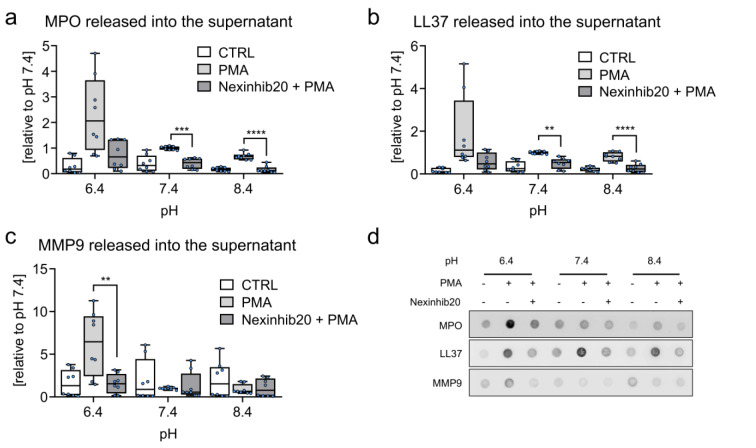
Inhibition of exocytosis in pH-cultured neutrophils by Nexinhib20 confirms pH-dependency of exocytosis. Primary neutrophils were cultured in different pH (6.4, 7.4, and 8.4) ± 100 nM PMA stimulation, ± Nexinhib20 (10 µM) for 3 h. Release of neutrophil granular proteins into the culture supernatant was detected by Dot Blot for a) Myeloperoxidase (MPO), b) Cathelicidin (LL-37), and c) Matrix-Metalloprotease 9 (MMP9); d) Representative Dot Blot images and the signal quantification by ImageJ. All quantified data were normalized to the pH 7.4 + PMA condition; N=4, n=2; Data are presented as Box plots with individual data points. Data were compared by 2-way-ANOVA, *p < 0.05, **p < 0.01, ***p < 0.001, ****p < 0.0001.

To determine whether the enhanced release of granule proteins at acidic pH resulted from increased exocytosis, experiments were repeated in the presence of the Rab27a-JFC1 interaction inhibitor Nexinhib20 (10 µM) (Johnson et al., 2016[20]). Inhibition of Rab27a significantly reduced the PMA-induced release of primary, specific, and tertiary granule proteins across all tested pH conditions (Figure 6a-d(Fig. 6)). Nexinhib20 only had no effect on the degranulation (Data not shown). Notably, the inhibitory effect of Nexinhib20 was strongest under acidic conditions, consistent with the pronounced degranulation observed in PMA-stimulated neutrophils at low pH.

### Alkaline pH favors polarization of primary granules

**Figure 7:**
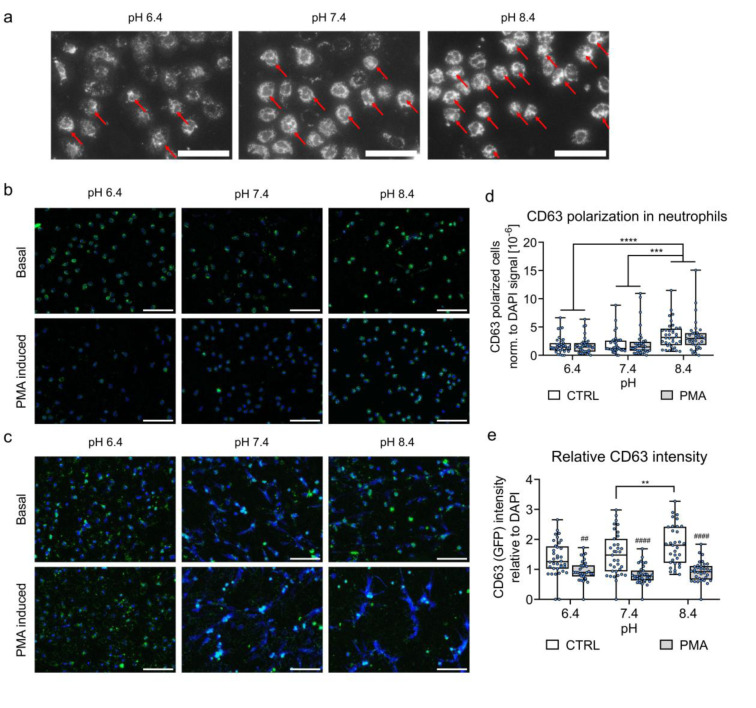
Changes of CD63 positive granules of primary neutrophils in different pH. a) Exemplary images of polarization effect (strong staining focused near the nuclei) observed in CD63-GFP stained, PMA induced neutrophils. Scale bar 50 µm. b-c) Representative images of CD63 (GFP) and Hoechst 33342 stained neutrophils ± PMA after 0.5 h (b) and 3 h (c) show polarization effect in alkaline pH (at 0.5 h) and the disappearance of the effect at 3 h. Scalebar: 100 µm. d-e) Increased number of relative CD63 polarized neutrophils in alkaline pH ± PMA. Stimulation of neutrophils with PMA results in reduced CD63-GFP signal intensity (normalized to Hoechst signal) independent of the pH. N=4; n=9; Data are presented as box plots with individual data points. Data were compared by 2-way-ANOVA, followed by multiple comparison, **p < 0.01, ***p < 0.001, ****p < 0.0001, #p < 0.05, ##p < 0.01, ###p < 0.001, ####p < 0.0001. * comparison between different pH and # comparison between basal and PMA induced in each pH.

To further evaluate pH-dependent effects on neutrophil degranulation, the subcellular localization of primary granules was examined by immunofluorescence staining for CD63 in neutrophils cultured at pH 6.4, 7.4, and 8.4, with or without PMA stimulation. In unstimulated cells, CD63-positive granules displayed a polarized distribution adjacent to the nucleus (counterstained with Hoechst 33342), with increasing fluorescence intensity from acidic to alkaline pH conditions. Within 30 minutes of PMA stimulation, CD63 staining redistributed toward the cell membrane under acidic and physiological pH conditions, indicating active granule mobilization. In contrast, under alkaline conditions (pH 8.4), CD63 polarization near the nucleus persisted, suggesting impaired exocytosis (red arrows, Figure 7a(Fig. 7)). Quantitative image analysis demonstrated a significantly higher proportion of CD63-polarized cells at pH 8.4 compared to pH 7.4 and 6.4 (Figure 7d(Fig. 7)). Moreover, total CD63-EGFP fluorescence intensity, normalized to DAPI staining, decreased following PMA stimulation and progressively declined over time, consistent with ongoing degranulation (Figure 7e(Fig. 7)). In line with earlier findings, NET formation was evident only in neutrophils cultured at physiological and alkaline pH, but not under acidic conditions (Figure 7c(Fig. 7)).

See also the Supplementary data.

## Discussion

The present study demonstrates a pH dependence of key neutrophil effector functions and reveals a distinct regulation of oxidative burst activity, NET formation, and degranulation by extracellular pH. While acidic conditions markedly suppressed ROS production and NET formation, neutrophil degranulation was strongly enhanced under the same conditions. In contrast, alkaline and physiological pH exerted little effect on exocytosis in primary human neutrophils, highlighting a differential pH sensitivity among neutrophil defense mechanisms.

Neutrophils are short-lived effector cells that serve as first responders in tissue injury and infection. Their half-life in the circulation is estimated to be approximately 10 h (Lahoz-Beneytez et al., 2016[23]), underscoring their capacity to rapidly adapt to changes in their microenvironment, such as pathogen invasion or fluctuations in ion concentrations. These properties are particularly relevant in the context of wound healing, where a rapid decline in tissue pH is observed immediately after injury (Schneider et al., 2007[42]). In contrast, chronic non-healing wounds are characterized by sustained alkalization (Wallace et al., 2019[47]). Given the early arrival of neutrophils at wound sites and their essential role in initiating the healing cascade, the present study focused on short-term neutrophil responses to pH changes. Consistent with previous reports, neutrophil viability was not compromised during three hours of incubation across a wide pH range (pH 6.1-8.4), aligning with studies that even describe anti-apoptotic effects of acidic conditions during prolonged incubation (Behnen et al., 2017[3]; Cao et al., 2015[8]).

One of the most potent antimicrobial strategies employed by neutrophils is the rapid generation of ROS during the oxidative burst, a central mechanism of innate immunity against bacterial and fungal pathogens (Nguyen et al., 2017[32]). In the present study, PMA stimulation robustly induced ROS production; however, the magnitude of the response was clearly pH-dependent. Acidic conditions significantly reduced both basal and PMA-induced ROS levels, in agreement with previous observations (Behnen et al., 2017[3]; Cao et al., 2015[8]; Khan et al., 2018[22]). In contrast to the findings of Khan et al., who reported increased ROS generation under alkaline conditions, no such effect was observed in our experimental setup. Differences in experimental design, stimulation protocols, or incubation times may account for these discrepancies. The attenuation of ROS production under acidic conditions may in part be explained by the increased concentration of protons, which can directly neutralize superoxide anions (Simchowitz, 1985[44]). Importantly, our data indicate that this effect requires sufficient time for cellular adaptation, as immediate pH shifts did not alter ROS levels. Once adaptation occurred, even basal ROS production was reduced at low pH. Given that NET formation induced by PMA, lipopolysaccharide, or bacteria relies on NOX-derived ROS (Remijsen et al., 2011[38]), the observed decrease in ROS provides a plausible explanation for the concomitant suppression of NET formation under acidic conditions. This interpretation is supported by earlier studies identifying ROS as a major trigger of NETosis and demonstrating reduced NET formation at low pH (Behnen et al., 2017[3]; Naffah de Souza et al., 2017[31]). Another contributing factor may be the pH-dependent regulation of antioxidant enzymes. GS levels were elevated under acidic conditions in unstimulated neutrophils, consistent with previous reports (Pietarinen-Runtti et al., 2000[34]). Upon PMA stimulation, levels of all analyzed antioxidant enzymes decreased, with GS and GPX1 exhibiting the most pronounced reduction in the alkaline pH range. A weakened antioxidant defense may enhance oxidative stress and thereby promote NOX-dependent NET formation. Brinkmann et al. recently identified SOD1 as a critical regulator of ROS production and NET formation, demonstrating that SOD1 activity is required for NETosis (Brinkmann et al., 2025[5]). Although this contrasts with the present findings, inhibition of SOD1 has also been associated with increased oxidative stress, which may be compatible with the observed pH-dependent shifts in ROS homeostasis. Given the detrimental role of excessive NET accumulation in wound healing (Zhu et al., 2021[50]), modulation of wound pH may represent a promising strategy to limit NET-associated tissue damage. However, reduced ROS production and NET formation may also compromise antimicrobial defense, emphasizing the need for a balanced regulation.

In addition to ROS generation and NET formation, neutrophils eliminate pathogens through the rapid release of antimicrobial and cytotoxic molecules stored in intracellular granules. ROS are known regulators of degranulation, as primary granules serve as reservoirs for NOX components and ROS can further stimulate granule release through protease activation and redox-dependent signaling pathways (Faurschou and Borregaard, 2003[13]; Potera et al., 2016[36]). Based on the reduced ROS levels observed under acidic conditions, an inhibitory effect on degranulation would have been expected. Surprisingly, acidic pH strongly enhanced PMA-induced exocytosis, particularly from primary and tertiary granules. To date, a dissociation between ROS production and enhanced degranulation has primarily been reported under hypoxic conditions (Hoenderdos et al., 2016[18]), where limited oxygen availability restricts ROS generation while promoting granule release via autophagy-related pathways (Lodge et al., 2020[27]). Under hypoxia, granule release occurs largely independent of granule subtype (Hoenderdos et al., 2016[18]). In contrast, the present data show preferential enhancement of azurophilic and gelatinase granule release under acidic conditions, suggesting that mechanisms beyond reduced ROS availability are involved. Neutrophil degranulation is a tightly regulated process governed by multiple signaling pathways. Protein kinase B (AKT), particularly the AKT2 isoform, has been described as a central intracellular switch controlling both ROS production and exocytosis. In murine models, AKT2 deficiency results in impaired ROS generation in neutrophils (Chen et al., 2010[10]). Behnen et al. reported increased levels of phosphorylated AKT under acidic conditions, which was associated with reduced apoptosis (Behnen et al., 2017[3]; Rane and Klein, 2009[37]). In addition to AKT signaling, the small GTPase Rab27a and its effector proteins play a pivotal role in neutrophil degranulation (Munafo et al., 2007[30]). The selective enhancement of primary and tertiary granule release observed here implicates Rab27a-associated effector proteins, such as synaptotagmin-like protein 1 (Slp1/JFC1) and Munc13-4, which govern granule docking and fusion. JFC1 downregulation selectively impairs MPO secretion from primary granules (Brzezinska et al., 2008[7]), whereas Munc13-4 is essential for tertiary granule release (Pivot-Pajot et al., 2008[35]). Accordingly, acidic pH may enhance degranulation by promoting the activity or interaction of these effector molecules with Rab27a, an interpretation supported by the effective blockade of acidic pH-induced exocytosis using the Rab27a-JFC1 inhibitor Nexinhib20. Consistent with this notion, immunofluorescence imaging revealed rapid polarization of azurophilic granules toward the nucleus following PMA stimulation, followed by redistribution toward the plasma membrane, except under alkaline conditions, which suppressed primary granule exocytosis. A comparable phenotype has been observed in Rac2-deficient neutrophils, which exhibit impaired release of primary and tertiary granules and persistent CD63 polarization (Ilarraza et al., 2023[19]). These similarities suggest a potential involvement of Rac2-dependent pathways in pH-regulated degranulation.

Several limitations of the present study should be acknowledged. Neutrophil viability was assessed only during short-term incubation, reflecting their limited lifespan *in vitro*; longer observation periods may reveal additional pH-dependent effects, including alterations in apoptosis. Furthermore, the pH-sensitive probe used primarily detected acidic shifts, while potential alkaline intracellular pH changes warrant further investigation. Finally, fluorescence signal variations likely originated predominantly from lysosomal compartments, which may explain the relatively modest changes observed.

## Conclusion

In summary, this study identifies extracellular pH as a key regulator of neutrophil effector functions. Acidic conditions suppress ROS production and NET formation while simultaneously enhancing neutrophil degranulation, particularly from primary and tertiary granules, indicating differential pH sensitivity of these defense mechanisms. Given the elevated pH and excessive NET accumulation characteristic of chronic wounds, targeted modulation of wound pH may represent a promising strategy to improve inflammation resolution and tissue repair.

## Declaration

### Conflict of interest

The authors declare no conflict of interest.

### Author contributions

Conceptualization: SE, MG; Methodology: MG, YL, FS; Validation: SE, AKN; Formal analysis: MG, MT; Investigation: MG; Resources: SE, AKN; Writing - original draft preparation: MG; Writing - review and editing, all authors; visualization; supervision: SE, AKN. All authors have read and agreed to the published version of the manuscript.

### Acknowledgments

The presented work is part of the dissertation of Maximilian Göbel. We would like to thank Kevin Schulz, Elisabeth Pfeffer, Leonie Tumminello, and Linnea Ceasar for their excellent technical support. We acknowledge support from the Open Access Publishing Fund of the University of Tübingen. 

### Artificial Intelligence (AI) - assisted technology 

Microsoft Co-Pilot was used for proofreading. The proofread version was carefully reviewed.

## Supplementary Material

Supplementary information

Supplementary data

## Figures and Tables

**Figure 1:**
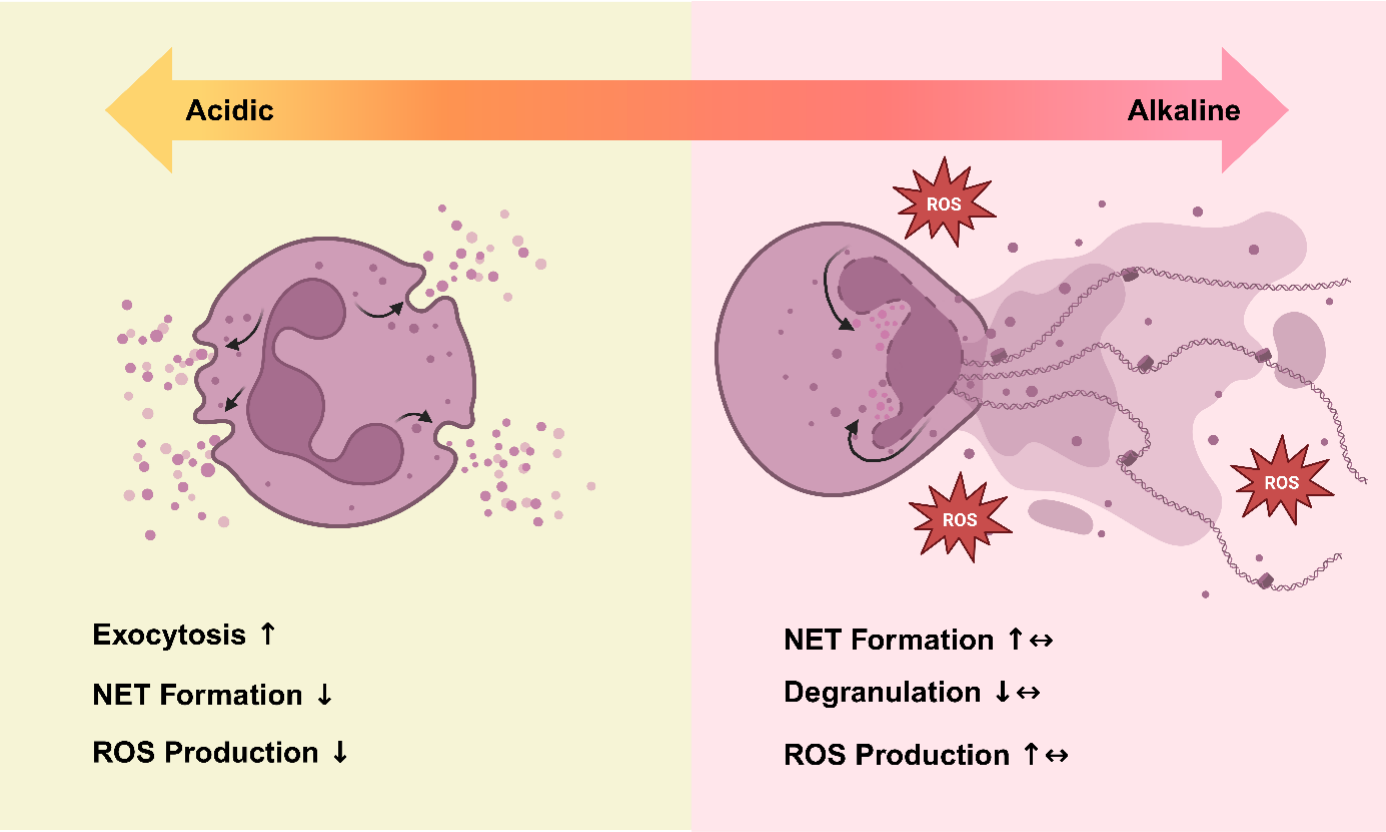
Graphical abstract
