# Supplementary material for: Acidic pH promotes degranulation and reduces oxidative stress in human primary neutrophils
Source: EXCLI J. 2026 Jul 13;25:1090–106. doi: 10.17179/excli2026-9092 (PMC13402789; doi:10.17179/excli2026-9092)
Supplement: Supplementary information [file EXCLI-25-1090-s-001.pdf]

EXCLI Journal 2026;25:1090-1106 – ISSN 1611-2156
Received: November 05, 2025, accepted: May 23, 2026, published: July 13, 2026

Supplementary information to:

Original article:

ACIDIC pH PROMOTES DEGRANULATION AND REDUCES OXIDATIVE STRESS IN HUMAN PRIMARY NEUTROPHILS

Maximilian Göbel, Yangfan Li, Melike Tombaz, Filiz Sahin, Andreas K. Nussler, Sabrina Ehnert*

Siegfried Weller Research Institute, BG Unfallklinik Tuebingen,
Department of Trauma and Reconstructive Surgery, Eberhard-Karls University Tuebingen,
Schnarrenbergstraße 95, D-72076 Tuebingen, Germany

* **Corresponding author:** Sabrina Ehnert, Siegfried Weller Research Institute, BG Unfallklinik Tuebingen, Department of Trauma and Reconstructive Surgery, Eberhard-Karls University Tuebingen, Schnarrenbergstraße 95, D-72076 Tuebingen, Germany. E-mail: Sabrina.Ehnert@med.uni-tuebingen.de

https://dx.doi.org/10.17179/excli2026-9092

This is an Open Access article distributed under the terms of the Creative Commons Attribution License (https://creativecommons.org/licenses/by/4.0/).

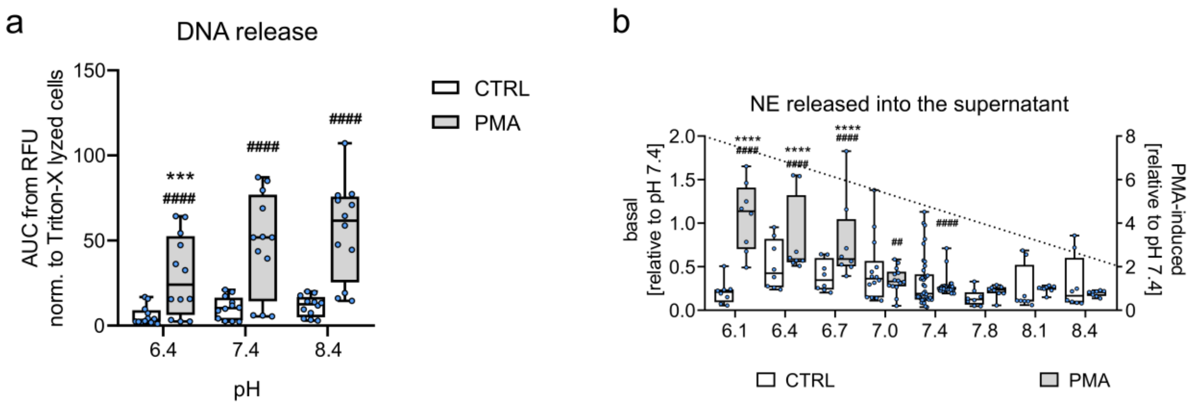

Supplementary Figure 1: a) Stimulation of primary neutrophils with 50 nM PMA results in a similar result as in 100 nM PMA stimulation. SYTOX Green Assay was used to determine DNA release. **b)** Protein of primary granules are higher released in acidic pH as shown with neutrophil elastase (NE). Protein amount was detected by Dot Blot method of neutrophils without PMA stimulation (white boxes - left axis) and 100 nM PMA stimulated (grey boxes - right axis). Data are presented as Box plots with individual data points; N=4, n=3 for **a)**, N=4, n=2 for **b)**, Statistical analysis was done by 2-way-ANOVA, while PMA-induced were compared to pH 7.4 respectively, ***$p < 0.001$, ****$p < 0.0001$. Differences in each pH from basal to PMA induced are marked by ##$p < 0.01$, ####$p < 0.0001$ (2-way-ANOVA).

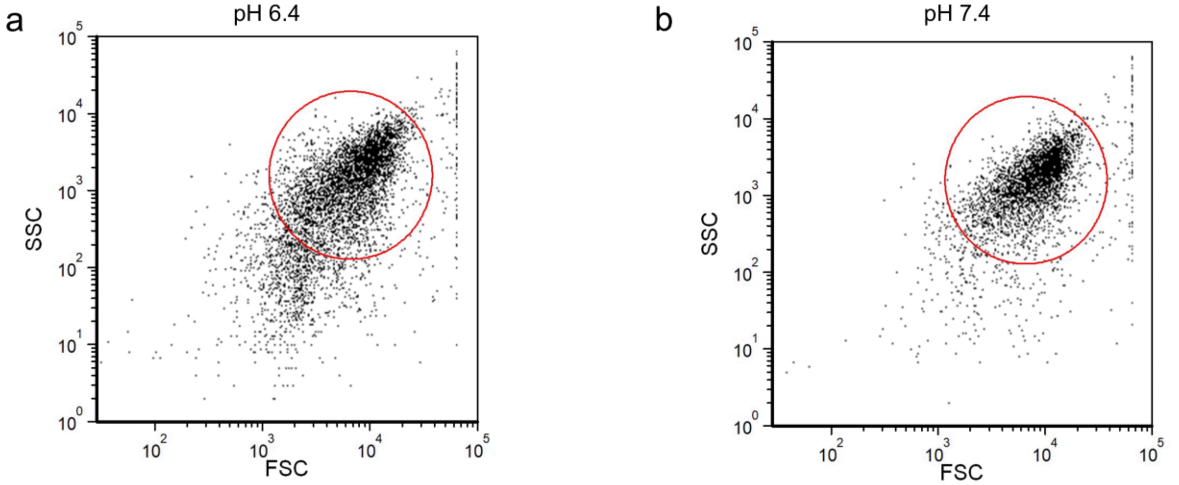

Supplementary Figure 2: Gating of the isolated neutrophils for single pH probe measurement (SPA chips CytoCHECK SPAchip® green single detection kit, S-001-PHG, a4cell nanodevices, OLS, Bremen, Germany. Only acidic pH (**a**) and physiologic pH (**b**) were measured.

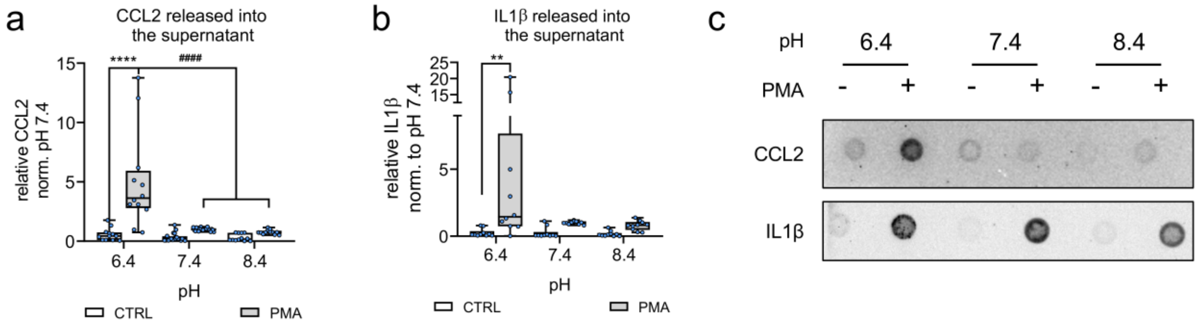

Supplementary Figure 3: Detection of neutrophil cytokine release in different pH by Dot Blot. Acidic pH results in stronger neutrophil cytokine release **a)** of CCL2, and in tendency of IL-1β **b)**, stimulation was done by 100 nM PMA (grey boxes). Exemplary Dot Blot membrane images in **c)**. Data are presented as Box plots with individual data points, N≥5, n=2, Statistical analysis was done by 2-way-ANOVA, while PMA-induced were compared to pH 7.4 respectively, ***p < 0.001, ****p < 0.0001. Differences in each pH from basal to PMA induced are marked by ##p < 0.01, ####p < 0.0001 (2-way-ANOVA).
